# GM-CSF Nitration Is a New Driver of Myeloid Suppressor Cell Activity in Tumors

**DOI:** 10.3389/fimmu.2021.718098

**Published:** 2021-10-05

**Authors:** Bianca Calì, Andrielly H. R. Agnellini, Chiara Cioccarelli, Ricardo Sanchez-Rodriguez, Andrea Predonzani, Giulia Ilaria Toffolo, Antonella Viola, Vincenzo Bronte, Giorgio Arrigoni, Francesco Zonta, Laura Albertoni, Claudia Mescoli, Ilaria Marigo, Barbara Molon

**Affiliations:** ^1^ Department of Biomedical Science, University of Padua, Padua, Italy; ^2^ Oncology and Immunology Section, Department of Surgery, Oncology and Gastroenterology, University of Padova, Padova, Italy; ^3^ Fondazione Istituto di Ricerca Pediatrica - Città della Speranza, Padova, Italy; ^4^ Veneto Institute of Oncology IOV-IRCCS, Padova, Italy; ^5^ Verona University Hospital, Department of Medicine, Verona, Italy; ^6^ Shanghai Institute for Advanced Immunochemical Studies, ShanghaiTech University, Shanghai, China; ^7^ Department of Medicine, Department of Medicine (DIMED), Surgical Pathology and Cytopathology Unit, University of Padua, Padua, Italy

**Keywords:** reactive nitrogen species, cytokines, immunosuppression, post-translational modification, tumor microenvironment

## Abstract

Reactive oxygen species, including RNS, contribute to the control of multiple immune cell functions within the tumor microenvironment (TME). Tumor-infiltrating myeloid cells (TIMs) represent the archetype of tolerogenic cells that actively contribute to dismantle effective immunity against cancer. TIMs inhibit T cell functions and promote tumor progression by several mechanisms including the amplification of the oxidative/nitrosative stress within the TME. In tumors, TIM expansion and differentiation is regulated by the granulocyte-macrophage colony-stimulating factor (GM-CSF), which is produced by cancer and immune cells. Nevertheless, the role of GM-CSF in tumors has not yet been fully elucidated. In this study, we show that GM-CSF activity is significantly affected by RNS-triggered post-translational modifications. The nitration of a single tryptophan residue in the sequence of GM-CSF nourishes the expansion of highly immunosuppressive myeloid subsets in tumor-bearing hosts. Importantly, tumors from colorectal cancer patients express higher levels of nitrated tryptophan compared to non-neoplastic tissues. Collectively, our data identify a novel and selective target that can be exploited to remodel the TME and foster protective immunity against cancer.

## Introduction

Host immune system can intercept, recognize and neutralize tumor cell clones. Following this paradigm, cancer immunotherapy has become a tangible opportunity for the treatment of different cancers as the metastatic melanoma, lung cancer and colorectal cancer (1, 2). In 2011, the US Food and Drug Administration (FDA) approved the first checkpoint-inhibitor drug (Ipilimumab) and nowadays a large number of clinical trials are ongoing worldwide. Nevertheless, immunotherapy still has obvious complexity and uncertainty. Indeed, the efficacy of such treatments is still incomplete mainly due to the unforeseen fatal side effects caused by the immune system overactivation and, more to the strong immunosuppression promoted by the engrafting tumors in the hosts. Seminal evidence indicates that tolerogenic and immunosuppressive circuits in tumor-bearing hosts co-opt host immune system finally dampening anti-tumor immunity. In particular, high frequency of myeloid immunosuppressive cells is tightly connected with tumor promotion, metastasis and poor prognosis in several type of cancers. Indeed, one of the key suppressive mechanisms tailoring cancer progression is the appearance of an unbalanced myelopoiesis in tumor-bearing hosts (3). This process leads to the expansion and differentiation of distinctive myeloid cell subsets that are principally involved in promoting T cell unresponsiveness toward tumor antigens (4, 5). The aberrant myelopoiesis occurring during tumor progression produce different types of immunosuppressive cells like tumor-associated macrophages (TAMs), tumor associated neutrophils (TANs) and myeloid derived suppressor cells (MDSCs). MDSCs are deeply investigated due to their strong ability to directly inhibit T cell functions and promote tumor progression. Early studies in tumor bearing mice described MDSCs as a heterogeneous population of myeloid cell lineage that is characterized by the concomitant expression of the surface markers CD11b and Gr-1 (6, 7). On the basis of phenotypical and functional features, MDSCs are currently arranged in two main subsets: polymorphonuclear MDSCs (PMN-MDSCs) and monocytic MDSCs (Mo-MDSC). In mice, PMN-MDSCs are phenotypically defined as CD11b^+^, Ly6C^low/-^, Ly6G^+^ cells while Mo-MDSCs are CD11b^+^, Ly6G^-^, Ly6C^hi^ (8). MDSCs were found in all types of cancer to a different extent. Nonetheless, their frequency and phenotype are extremely variable in human malignancies thus increasing the complexity of their characterization. Key drivers for MDSC generation and expansion are two cytokines: the GM-CSF and IL-6. Indeed, clear evidence in transplantable tumor models unequivocally connects GM-CSF to the systemic expansion of an immature myeloid population (MDSCs) that actively suppresses anti-tumor T cell immunity (9–11). *In vivo* data confirmed that tumor-derived GM-CSF orchestrates the intra-tumoral accumulation of highly immunosuppressive CD11b^+^Gr-1^+^ myeloid cells in a spontaneous pancreatic ductal adenocarcinoma (PDAC) model (12). Nonetheless, other experimental evidence paradoxically indicates that GM-CSF acts as a potent immunostimulatory product and its efficacy as vaccine-adjuvant has been demonstrated in different regimens (13, 14). Reasonable explanations for this antithetical conduct could be found in a quantitative difference in location and dosage of the very same cytokine in tumors (15) or most likely, in a qualitative divergent tumor-related mechanism. Thus, GM-CSF can act in cancer as corrupted cytokine, co-opted by the tumor itself to sculpt an immunosuppressive environment ultimately establishing its immune privilege (16).

Notably, the release and overload of inflammatory reactive species are prominent features of cancer. In particular, high levels of nitrated proteins have been detected in several human tumors, including prostate, colon, liver, breast, and ovarian neoplastic tissues (17). Our group previously discovered a novel mechanism of tumor escape based on the post-translational modification (PTM) of intra-tumoral chemokines, which can be pharmacologically targeted to improve the efficacy of cancer immunotherapy. Indeed, the nitration of the inflammatory chemokine CCL2 within the tumor microenvironment (TME) altered its chemoattractant properties finally limiting T cell entry into the primary tumor lesion (18). Although we originally focused on a single protein, other cytokines and chemokines should be target of such modification contributing to shape anti-tumor immunity. In this manuscript, we reasoned that the cytokine GM-CSF, which is abundantly expressed in several human cancers, represents a possible target for ROS/RNS-induced PTM in the TME. We found that RNS significantly altered the GM-CSF activity in tumor-bearing hosts by causing protein nitration at a specific site, a tryptophan, that we identified. Moreover, we revealed the molecular signature activated by the nitrated cytokine in myeloid cells, which is responsible for their enhanced immunosuppressive phenotype and activity. Finally, we provided evidence that GM-CSF nitration *in vivo* significantly affects the immune landscape at the tumor site. Importantly, GM-CSF nitration in the Tryptophan residue can be also found in human colorectal cancer specimens, where we observed higher levels of both GM-CSF and nitrated-Tryptophan in comparison to the surrounding non-transformed ones.

Collectively, our data identify the nitrated-GM-CSF as a novel player of tumor-related immunosuppression, which can be pharmacologically targeted in novel immune-based strategies against cancer.

## Methods

### Mice

C57BL/6 and OT-I (C57BL/6- Tg (Tcra Tcrb) 1000Mjb/J mice were purchased from Charles River Laboratories. All experiments were performed with mice of 8 weeks maintained in the animal facilities of the Venetian Institute of Molecular Medicine (VIMM). Experiments were performed according to the guidelines approved by the local ethics committee.

### Plasmids

The encoding sequence for GM-CSF were taken from pubmed and obtained by gene synthesis from GenScript. This sequence codifies the full length of GM-CSF plus the 6XHis tag introduced at the C-terminus after AgeI restriction site. To allow further cloning, two cloning sites were introduced at 5’ and 3’. The plasmid obtained from GeneScript were cloned HINDIII/NHE into pcDNA3.1-hygro (Invitrogen). To generate the mutant version of GM-CSF, a site-specific mutagenesis approach was used with the following oligos:

mut W>L for: 5’-ccatcactgtcacccggcctCTgaagcatgtagaggccatc-3’
mut W>L rev:5’-gatggcctctacatgcttcAGaggccgggtgacagtgatgg-3’.

### Cell Line

MCA-203 fibrosarcoma cell line was maintained in DMEM (Lonza) supplemented with 10% heat-inactivated FBS (Gibco), 2 mM L-glutamine (Gibco), 10 mM HEPES (Lonza), 55 uM 2-mercaptoethanol (Gibco), 150 U/ml streptomycin, 200 U/ml penicillin obtained from the American Type Culture Collection (ATCC). Two additional cell lines were generated by the stable transfection with either electroporation or the usage of Lipofectamine 2000 (Invitrogen) of MCA cell line with the plasmid encoding either GM-CSF or its mutant version GM-CSF W30L. Transfected cells were selected and thus culture in the media used for MCA-203 supplemented with 0,3 ug/ml Hygromicin (Invitrogen).

### Tumor Challenge

C57BL/6 mice were injected on the right flank with 10^6^ MCA203-GM cells or MCA203-W30L cells. Tumors were measured every 2 days by a digital caliper. At day 21 post-tumor challenging, mice were euthanized, and their tumors explanted and processed for flow cytometry or immunofluorescence analysis

### Tumor Dissociation

Explanted tumors were cut in small pieces with a scissor; pieces were covered with a digestive solution composed of collagenase IV (1 mg/ml) hyaluronidase (0,1 mg/ml) and DNase (0,03 KU/ml) and incubated at 37°C; every 10 minutes tumors were mechanically processed using a 5 ml pipette. After 1 hour, cells were collected and washed twice in complete medium to remove all digestive solution.

### Cytokine Nitration

Recombinant mouse GM-CSF was purchased from Miltenyi Biotech (130-095-742). Cytokine nitration was performed by adding 1mM peroxynitrite (Millipore) to the recombinant protein at 37°C for 15 min in a final volume of 100ul PBS (Lonza). After incubation, the samples were extensively dialyzed in PBS for 5 hours using the Slide-A-Lyzer Dialysis cassette kit, 3,500 MWCO (Thermo Fisher Scientific). Finally, cytokines were collected and used for *in vitro* assays at 20 ng/ml.

### LC-MS/MS Analysis

Equal amounts of recombinant GM-CSF(1 µg) either untreated or treated with peroxynitrite or with degraded RNS were digested using sequencing-grade modified trypsin (Promega) with a protein/enzyme ratio of 50:1, as described in (18). Peptides were injected into a 10 cm pico-frit capillary column (75 μm, I.D. New Objective) packed in house with C_18_ material (ReproSil, 300A°, 3 μm, Dr. Maisch GmbH) using a Ultimate 3000 HPLC system (Dionex – Thermo Fisher Sicentific) and separated using a linear gradient of acetonitrile/0.1% formic acid from 3% to 40% in 15 min at a flow rate of 250 nl/min. Eluted peptides were analyzed using a LTQ-Orbitrap XL mass spectrometer (Thermo Fisher Scientific) coupled online with the LC system through a nano-spray ion source (Thermo Fisher Scientific). The MS system operated in a data dependent mode, by acquiring a full MS scan at high resolution in the Orbitrap analyzer, followed by the MS/MS scans in the linear Ion Trap of the 10 most intense ions. Data were searched using Mascot Search Engine (Matrix Science) against the SwissProt database (Version 2013_04, 539829 sequences; 191670831 residues). Precursor and fragment ion tolerance were set to 10 ppm, and 0.6 Da respectively. An error tolerant search was done to highlight possible modifications and nitration of Tryptophan 30 (W30) was identified as one of the main post-translational modifications, which was present at high concentration only in the sample treated with peroxynitrite. The mass spectrometry proteomics data have been deposited to the ProteomeXchange Consortium *via* the PRIDE (19) partner repository with the dataset identifier PXD027049.

### Bone-Marrow Derived MDSC

C57BL/6 mice were sacrificed, tibias and femurs were removed to flush the bone-marrow under sterile conditions. Red cells were lysed with ACK Lysing Buffer (Lonza). To obtain MDSCs 2x10^6^ cells were plated into 6-well plate in medium supplemented with 20 ng/ml of GM-CSF (Miltenyi) native or nitrated by peroxynitrite (Millipore) in combination with 20 ng/ml of IL-6 and cultured for 5 days at 37°C in 5%CO_2_.

### Cytofluorimetric Analysis

Cells derived from *in vitro* bone marrow differentiation or cells isolated from tumor-bearing mice, were blocked with FcR binding sites and stained with the following antibodies: anti-CD11b (clone M1/70), anti-Ly6G (clone 1A8), anti-F480 (clone CI:A3-1), anti-Ly6C (clone HK1.4), anti-pStat3, anti-p-Stat5, anti-p-Erk, anti-CD45.1 (cloneA20). Aqua Live/Dead^®^dye (Invitrogen) was used to analyze cell viability. Intracellular staining was performed using BD Cytofix/Cytoperm Kit in association with BD Phosflow Perm Buffer III. Flow data were acquired with FACSAriaII and analysed with FlowJo software (Tree Star, Inc.).

### Immunofluorescence

Mouse tumors were collected and fixed in 4% paraformaldehyde, cryoprotected in 30% sucrose and frozen in OCT. The samples were cut with cryostat (7μm), incubated with blocking solution (10% FBS, 3% BSA) 2 hours at room temperature. The following primary antibodies were used: anti-nitrotyrosine (Millipore), anti-nitrotryptophan (JaICA), anti-F480 (Abcam), anti-CD31 (R&D Systems). The appropriate secondary antibodies were used. For quantitative analysis, 10 different and non-contiguous regions of interest (ROIs) were randomly selected, and percentage of positive areas was obtained for each marker. Images were acquired with a Leica TCS SP5 confocal microscope. Images and colocalization indexes were analyzed by ImageJ Software.

### Immunohistochemistry

Human colorectal specimens (n=6) were obtained from the Padua Hospital in accordance with the ethical standards. This study was examined and approved by the Ethics Committee of the local Health and Social Services (Azienda Ospedaliera, Padova) in accordance with the ethical standards laid down in the 1964 Declaration of Helsinki. Specimens were frozen in OCT, cut with cryostat (7µm) and analysed with doublestain IHC Kit (Abcam) according to manufacturer’s instructions. For quantitative analysis 10 different and non-contiguous regions of interest (ROIs) were randomly selected, and percentage of positive areas was obtained for each marker. Images were acquired by Olympus IX81and analyzed by ImageJ Software.

### RNA Purification and Real-Time PCR Analysis

To confirm the presence of GM-CSF encoding sequences, RNA extraction was performed using RNeasy Mini Kit (QIAGEN). cDNA was obtained with SuperScript II reverse transcriptase and poly dT primers (Invitrogen). PCR was performed in a volume reaction of 10ul containing TaqMan Universal PCR Master Mix (Applied Biosystem) and 20 ng cDNA with the following couple of primers (5’ – 3’ etc). We used Applied Biosystems 7900HT-Fast Real-time for PCR reaction and fluorescence detection. All measures were performed in duplicates and were analyzed by the ΔΔCt relative quantification method: arithmetic means of the cycle threshold (Ct) values were calculated and target gene mean Ct values were normalized to the respective endogenous control (*Gapdh*). The values obtained were converted by the formula 2(−ΔΔCt) to be expressed as fold changes in regulation compared with the reference sample.

### Mixed Lymphocyte Peptide Culture and Proliferation Assay

MLPC cultures were prepared by mixing γ-irradiated C57BL/6 splenocytes with OT-I/CD45.1 splenocytes in order to obtain 1% OVA-specific T lymphocytes in the final culture. OVA-specific lymphocytes were previously CFSE-labeled (carboxyfluorescein succinimidyl ester, Cell Trace Kit, Invitrogen Molecular Probe) according to manufacturer’s instructions for the proliferation assay. A total of 0.6x10^6^ mixed cells were plated in flat-bottom 96-well plates and stimulated for 3 days with 1 μg/ml of OVA peptide (OVA_257-264_, SIINFEKL). Where required, MDSCs (derived from immunomagnetic sorting) were added at decreasing percentages (12% and 6%). T Cell proliferation was evaluated by FACS and data analyzed by FlowJo software (Tree Star, Inc.) using the *FlowJo’s Proliferation* Tool by tracking cell generation according to software indications.

### ELISA

The GM-CSF concentrations in cell culture supernatants and in serum from mice were evaluated using mouse GM-CSF Quantikine ELISA kit (MGM00 R&D System, Minneapolis, MN) according with manufacturer’s instructions.

### Western Blot

Total protein extract was obtained with RIPA buffer. Protein extracts were separated by 4-12% Bold NuPage (ThermoScientific) and transferred onto PVDF membranes (BioRad). After blocking with 3% albumin (Sigma-Aldrich) and primary antibody incubation phospho-STAT5, phospho-Erk1/2, Erk1/2 -Cell Signaling- and STAT5 -Santa Cruz- 1:1000), the membranes were incubated with an anti-rabbit peroxidase-conjugated secondary antibody (GE healthcare). Chemiluminescence was obtained by the ICL Substrate (GE healthcare), and images were obtained with an imaging ImageQuant LAS 500 (GE healthcare).

### Statistical Analysis

Statistical comparison between two groups was carried out using the Student’s T test or Mann Whitney test. For multiple comparisons, One-way Anova test, followed by a Tukey post-hoc test was performed. Results with a p-value ≤ 0.05 were considered as significant. In figures, asterisks were used as follows: *p ≤ 0.05; **p ≤ 0.01; ***p ≤ 0.001; ****p ≤ 0.0001. Analyses were performed using Graph Pad Prism (version 7).

## Results

### Cytokine Nitration Affects Myeloid Cell Commitment by Activating Multiple Signaling Pathways

**Figure 1:**
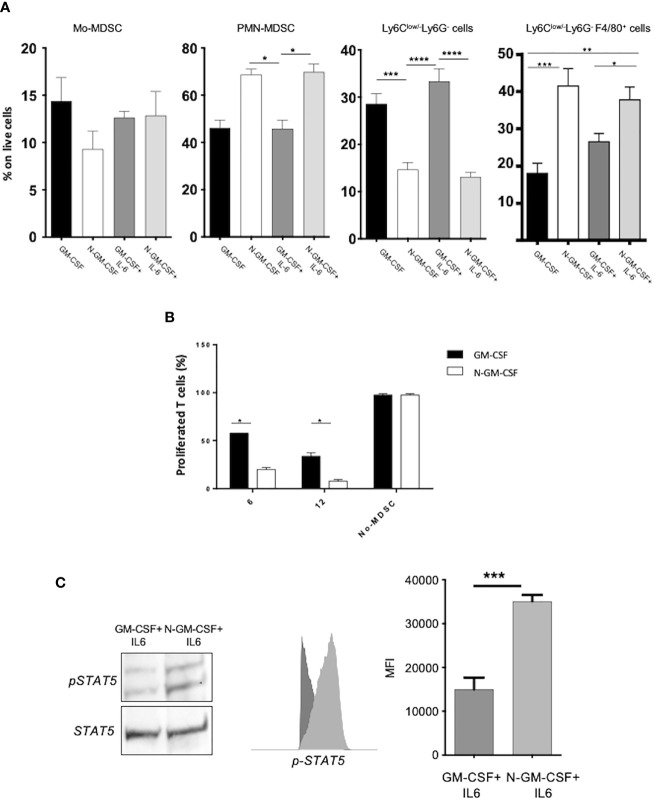
GM-CSF nitration boosts myeloid cell commitment and activates immunosuppressive-related signaling pathways. **(A)** Bone-marrow progenitors were cultured in the presence of either untreated GM-CSF or with peroxynitrite-treated GM-CSF (N-GM-CSF) alone or in combination with recombinant IL-6. The Bar graph represents the percentage of cells for each subset on CD11b^+^ myeloid cells after 4 days of culture. Data represent means ± s.e.m from 6 independent experiments; *p ≤ 0.05; **p ≤ 0.01: ***p ≤ 0.001) **(B)** Different percentages (6-12%) of BM-MDSCs obtained after 5 days of culture with either GM-CSF + IL-6 or N-GM-CSF + IL-6 were tested for their ability to suppress the proliferation of OVA-specific, OT-I CD8^+^ T cells stimulated by the SIINFEKL peptide in culture. Bar graph represents the percentage of proliferating T cells. OT-I CD8^+^ T cells stimulated with peptide in the absence of BM-MDSC were used as control. **(C)** Representative WB and FACS analysis of the phosphorylation level (expressed as MFI) of STAT5 protein in BM-MDSCs differentiated by GM-CSF or N-GM-CSF or in combination with IL-6. Data represent means ± s.e.m from 4 independent experiments. Significance was determined by one-way Anova (Tukey test for multiple comparisons; *p ≤ 0.05; **p ≤ 0.01: ***p ≤ 0.001; ****p < 0.0001).

GM-CSF is commonly up regulated in multiple human tumors where it exerts a recognized tumor-promoting action (20). Nonetheless, evidence indicates a supportive role for this cytokine in fostering anti-tumor immunity (21). In a previous report, we revealed that the nitration of the CCL2 chemokine, caused by the oxidative overload within the tumor mass, impacted on immune cell trafficking by selectively affecting anti-tumoral T cell infiltration (18). Here, we reasoned that post-translational modification (PTM) could be responsible for the antithetical course of action of GM-CSF in tumors. To test our hypothesis, we at first assessed whether GM-CSF represents a ROS/RNS target by timely exposing the recombinant (human and mouse) GM-CSF to commercial peroxynitrite (ONOO^-^); the presence of reproducible modifications as nitration/nitrosylation and/or oxydation was evaluated by tandem mass spectrometry coupled to liquid chromatography (LC-MS/MS). Our results indicated that a single Tryptophan residue, in the SPITVTRPW*K peptide was stably nitrated, as demonstrated by a +45Da shift in the tryptophan residue (where * represents nitration, +45 Da) upon peroxynitrite treatment (**Supplementary Figure 1A**). The accumulation of myeloid immunosuppressive cells (MDSCs) at the tumor site correlates with bad prognosis in different type of cancers (22) and it has been well-established that GM-CSF acts as driver for MDSC differentiation in tumors (12). To investigate the effect of GM-CSF nitration on MDSC commitment and functions, we generated *in vitro* fully competent MDSCs from bone marrow precursors (BM-MDSCs) by adding GM-CSF and IL-6 cytokines as previously published (11). In particular, we used ONOO^–^treated GM-CSF (called hereafter N-GM-CSF) or GM-CSF treated with degraded ONOO^-^ (control) alone, or in combination with IL-6. After 5 days of culture, we phenotypically profiled harvested culture cells by multiparametric flow-cytometry (FACS). Interestingly, FACS analysis revealed a higher expansion of the PMN-MDSC subset in cells differentiated in presence of N-GM-CSF, alone or in combination with IL-6, in comparison to cells differentiated with the unmodified cytokine. No significant differences were observed for the Mo-MDSC subset. Moreover, N-GM-CSF exposure induced an increment of macrophages (Ly6C^low/-^ Ly6G^-^ F4/80^+^) and a concomitant reduction of Ly6C^low/-^Ly6G^-^ cells (**Figure 1A** and **Supplementary Figure 1B**). Further, we evaluated the effect of N-GM-CSF on the immunosuppressive functions of BM-MDSCs. OVA specific CFSE-labeled T lymphocytes were stimulated to proliferate by adding the specific OVA-peptide to the culture, in the presence or not of different concentrations of BM-MDSCs, previously differentiated with either N-GM-CSF+IL-6 or GM-CSF+IL-6 cytokines. We observed that MDSCs obtained from the N-GM-CSF+IL-6 cultures were more efficient in inhibiting CD8^+^ T lymphocyte proliferation compared to the GM-CSF/IL-6 cultures (**Figure 1B**). Our results indicated that the biological activity of GM-CSF is significantly altered by protein nitration. Indeed, N-GM-CSF sustains *in vitro* the expansion of myeloid cell subsets endowed with higher immunosuppressive activity as compared to the unmodified cytokine.

We next sought to investigate the molecular pathways triggered in myeloid cells upon N-GM-CSF stimulation. It is known that GM-CSF interaction with its receptor, the GM-CSFR, activates the Erk1/2 kinases and downstream selected transcriptional factors including among others, STAT5, which in turn regulates cell proliferation, differentiation and survival (23). In particular, STAT5 phosphorylation has been linked to T cell suppression by promoting apoptosis and Erk1/2 kinases was associated with myeloid precursors differentiation (4, 24). We found that N-GM-CSF, in combination with IL-6, induced a significant increment in the phosphorylation level of STAT5 and Erk1/2 in N-GM-CSF-derived MDSCs, as compared to the unmodified cytokine, (**Figure 1C** and **Supplementary Figure 2**). Collectively, our data suggested that the nitration of GM-CSF impinges on myeloid cell commitment and immunosuppressive activity possibly boosting the STAT5 and MAPK pathways in differentiating BM-precursors.

### N-GM-CSF Affects Myeloid Cell Commitment and Activity in Tumor-Bearing Hosts

To specifically address the impact of RNS-modified GM-CSF in tumor progression, we genetically modified the murine fibrosarcoma cell line MCA-203, that did not secrete *per se* detectable levels of GM-CSF both *in vitro* and *in vivo*. Specifically, we generated tumor-cell clones stably expressing either a plasmid encoding the full sequence of the murine GM-CSF (named GM) or a full-mutant sequence (named W30L), where we introduced a point mutation at the site of protein nitration. In more detail, we substituted the RNS-targeted amino acid residue (tryptophan, W) with a no-targetable one, by a site-specific mutagenesis approach. We changed the codon encoding W in position 30 of murine GM-CSF with the one for leucine (L). This substitution was driven by a bioinformatic analysis where different homologues and orthologues protein sequences where aligned. Interestingly, our analysis indicated that this position is well conserved, with very few cases where other amino acids are encoded. Moreover, the codon for leucine has the highest substitution score according to two independent substitution matrix (PAM250 and Blosum) and is not known to be sensitive to nitration/nitrosylation such as other aromatic residues or polar residues (25) (**Supplementary Figures 3A, B**). Computational modelling analysis of murine GM-CSF structure confirmed that the tryptophan in position 30 (W30) is exposed to the solvent and is a good candidate for protein nitration. This analysis also highlighted the stability of the W30L mutant (**Supplementary Figure 3C**). By this strategy, we obtained multiple MCA-203 clones expressing either the GM or the W30L plasmids. Among them, we selected a pair of clones, hereafter named GM and a W30L, which displayed, *in vitro*, the same ability to proliferate (data not shown) and that were able to secrete comparable amount of GM -CSF (**Supplementary Figure 4A**). Supernatants from GM and W30L clone cultures were tested for their ability to differentiate *in vitro* MDCSs from BM precursors. Recombinant GM-CSF and IL-6 were used as controls. We did not observe differences in the percentage and morphology of MDSC subsets (Mo-MDSC, PMN-MDSC and Ly6C^low/-^ Ly6G^-^) obtained from BM cell cultures upon the stimulation with either MCA203-GM or MCA203-W30L secretome. However as expected, cell supernatants were less effective than the recombinant cytokines in differentiating myeloid cell populations (**Supplementary Figures 4B, C**).

**Figure 2:**
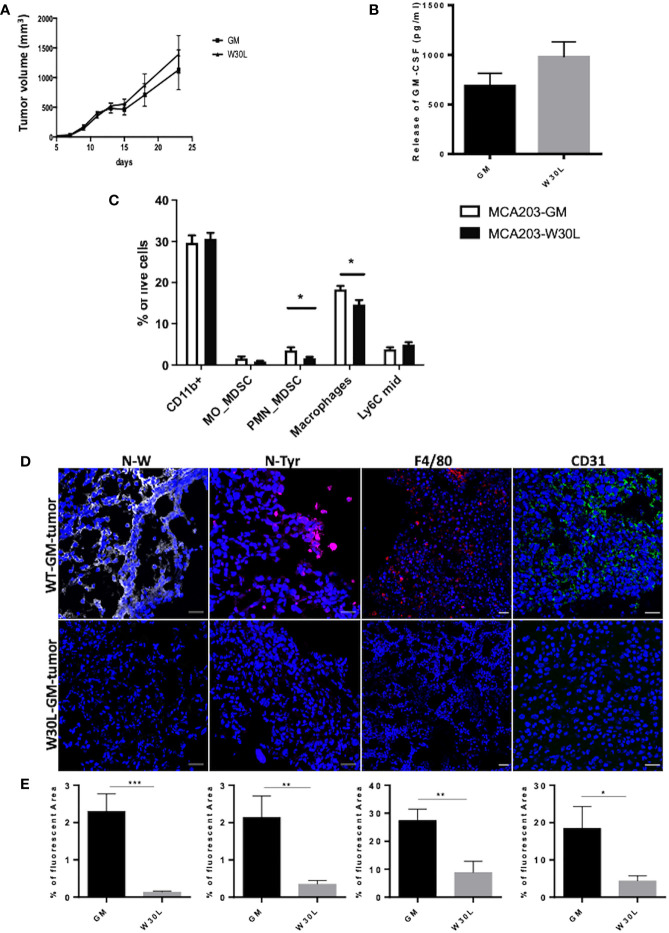
The nitration of GM-CSF impacts on myeloid cell frequency within the tumor microenvironment **(A)** C57BL6 mice were s.c challenged with either MCA203-GM or MCA203-W30L tumor cell clones. Tumor volume was measured at the indicated time-points; **(B)** At day 21 after tumor injection the amount of GM-CSF in serum of mice was quantified by ELISA. **(C)** Frequency of myeloid subsets on live cells in tumor tissues. Error bars represent means ± s.e.m; data are representative of 3 independent experiments unpaired Student t test analysis (*p ≤ 0.05) **(D)** Representative images of IF of tissue slices from either MCA203-GM or MCA203-W30L tumors. Tissues were stained for nitro-tryptophan (grey), nitro-tyrosine (magenta) F4/80 (red) or CD31 (green). **(E)** Quantification of fluorescence (positive reactive areas) from nitro-tryptophan, nitro-tyrosine, F4/80, CD31 staining. Scale bar, 25 μm. Data represent means ± s.e.m from 3 independent experiments; unpaired Student T-test *p ≤ 0.05; **p ≤ 0.01: ***p ≤ 0.001.

To dissect the impact of GM-CSF nitration *in vivo*, we challenged immunocompetent C57BL/6 mice with either MCA203-GM or MCA203-W30L tumor cells. We did not observe significant differences in the tumor growth between the two engrafted clones (**Figure 2A**). Moreover, we detected comparable amounts of circulating GM-CSF in the serum of mice injected with the two different tumor clones (**Figure 2B**). Three weeks later the MCA-203 challenging, we analyzed myeloid cell accumulation at the tumor site. We did not find any significant variations in the total percentage of CD11b^+^ cells (around 30% of intra-tumoral MDSCs on total tumor cells) but we observed a significant reduction in the percentage of PMN-MDSCs (Ly6G^+^) and macrophages (Ly6C^-^/Ly6G^-^/F4/80^+^) in mice MCA203-W30L-bearing mice compared to the MCA203-GM controls (**Figure 2C** and **Supplementary Figure 5A**). These data suggested that the nitration of the GM-CSF amplifies the recruitment and commitment of immunosuppressive myeloid cells in tumors.

We also evaluate the level of N-W at the tumor core by confocal microscopy and, as expected we found a significant reduction in tryptophan nitration in W30L tumors compared to GM controls. At the same time, we detected a significant reduction in the nitro-tyrosine content in MCA203-W30L tumors suggesting that the nitration of GM-CSF locoregionally tuned the recruitment and expansion of cell subsets that can sustain the oxidative/nitrosative stress at the tumor core (**Figures 2D, E** and **Supplementary Figure 5B**). Evidence indicated that GM-CSF also promotes immune-independent tumor progression by directly supporting cancer cell survival and tumor angiogenesis (26). We observed a consistent decrease in the endothelial marker CD31 in W30L tumors compared to GM controls (**Figures 2D, E** and **Supplementary Figure 5B**), suggesting that GM-CSF nitration might also affect tumor angiogenesis.

**Figure 3:**
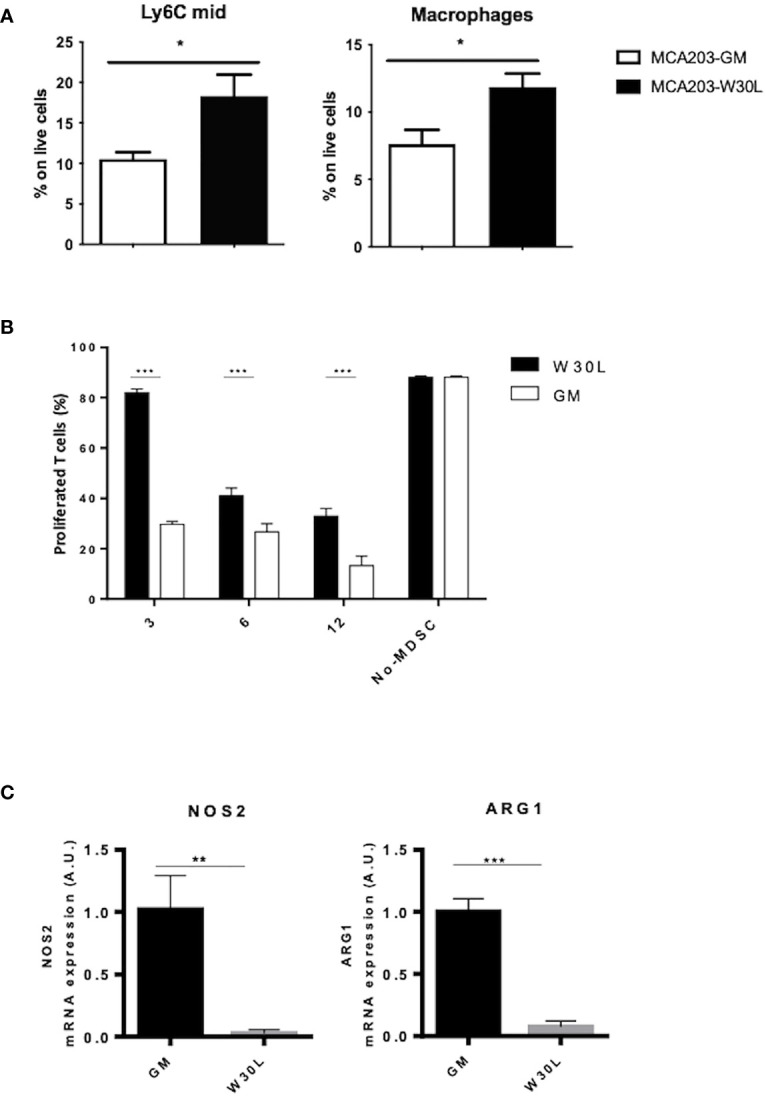
The nitration of GM-CSF affects the differentiation and activity of myeloid cells in tumor-bearing hosts. **(A)** C57BL6 mice were s.c. challenged with either MCA203-GM or MCA203-W30L tumor clones. At day 21, after tumor challenge mice were sacrificed and myeloid cell subpopulation were quantified by FACS. **(B)** CD11b^+^ cells magnetically-purified from the spleen of either MCA203-GM or MCA203-W30L tumor-bearing mice were co-cultured with OVA-specific, CFSE-labeled, CD8^+^ T lymphocytes in the presence of OVA peptide. Bar graph shows the percent of proliferating T cells. Lymphocytes stimulated with OVA peptide in absence of myeloid cells (no-MDSC) were used as controls. **(C)** NOS2 and ARG1 mRNA expression was evaluated by RT-PCR Data represent means ± s.e.m from 3 independent experiments; unpaired Student T-test *p ≤ 0.05; **p ≤ 0.01: ***p ≤ 0.001.

The spleen plays a crucial role during tumor progression since it represents the elective site for the induction of tumor tolerance mediated by myeloid cells (27). Indeed, we moved to characterize myeloid cell landscape in the spleen of either MCA-GM or W30L-GM tumor-bearing mice. We found a significant increase of immature monocytes (Ly6C^mid^) and macrophages in the spleens of W30L tumor bearing mice as compared to the control (**Figure 3A**). To evaluate the functional phenotype of these myeloid cell populations, we isolated CD11b^+^ cells from the spleens of either MCA203-W30L or MCA203-GM-bearing mice, and we assessed their immunosuppressive activity by co-culturing CD11b^+^ with antigen-activated T lymphocytes. Remarkably, we observed that MDSCs from W30L tumors were less efficient in suppressing T cell proliferation in comparison to the GM controls (**Figure 3B**). Finally, as MDSC immunosuppressive potential in tumors mainly rely on the expression and activity of two key enzymes, Arginase 1 (ARG1) and Nitric Oxide Synthase 2 (NOS2), we checked by Real time-PCR the transcription level of these enzymes in CD11b^+^ cells isolated from the spleens of tumor-bearing mice. Importantly, we found that myeloid cells from MCA203-W30L tumor-bearing mice expressed lower level of both ARG1 and NOS2 enzymes as compared to the control (**Figure 3C**), that could potentially explain their reduced suppressive activity. Collectively, our findings indicated that the nitration of GM-CSF deeply conditions tumor immune contexture and more, it boosts the systemic expansion and activity of MDSC in tumor bearing hosts.

### Tryptophan Nitration in Colorectal Cancer

**Figure 4:**
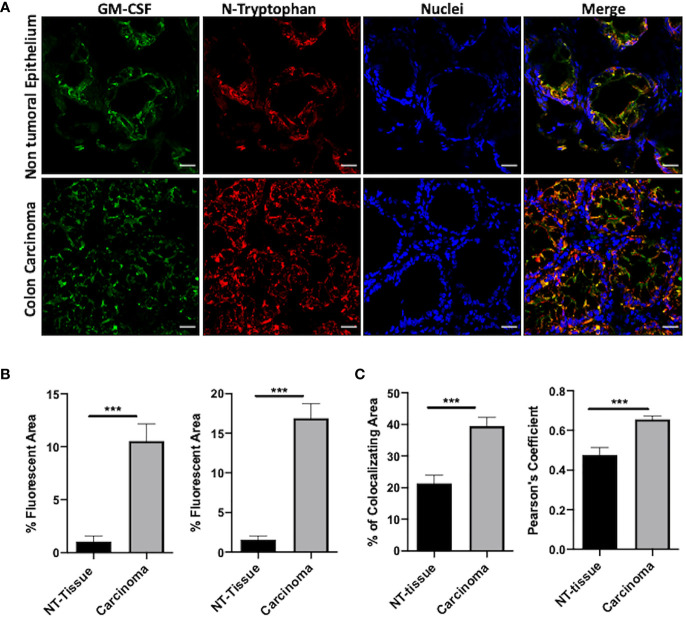
N-GM-CSF in human colon carcinomas. **(A)** IF images of a specific area in human colon carcinoma samples. Blue: nuclei; red: nitro-tryptophan, green: GM-CSF; yellow:merge; **(B)** Quantification of fluorescence (positive reactive areas) for GM-CSF and nitro-tryptophan staining. Scale bar, 25 μm. **(C)** Percentage of reactive areas where nitro-tryptophan and GM-CSF co-localized in either carcinomas or NT tissues (upper graph). Pearson Coefficient of co-localization for nitro-tryptophan and GM-CSF in either carcinomas or NT-tissues (bottom graph). Data are representative of n = 6 patient biopsies, unpaired Student T-test analysis (***p < 0.001).

The expression of GM-CSF in human colorectal primary tumors has been extensively documented. Interestingly, elevated levels of soluble GM-CSF have been recognized in the serum of colorectal cancer patients, suggesting that GM-CSF may be an independent prognostic factor (28). However, the role of cytokine in colorectal cancer still needs to be defined being GM-CSF endorsed with either tumor -suppressing or tumor-promoting activity in human patients (20, 29). Indeed, we assessed the expression of GM-CSF in colorectal primary tumor specimens and we observed that GM-CSF is more abundant at the core of the lesion in comparison to the surrounding non tumoral epithelium (NT-tissue) (**Figures 4A, B** and **Supplementary Figures 6SA, B**). We next evaluated the presence, the distribution and the abundance of nitrated-Tryptophan (N-W) in the same tumor specimens by using an antibody specifically recognizing nitro-tryptophan residues. Our analysis revealed a significant increment of N-W at the neoplastic foci compared to the surrounding not-transformed tissue (**Figures 4A, B** and **Supplementary Figures 6SA, B**). The level of the oxidative/nitrosative level in the very same tissues was also confirmed by the nitro-Tyrosine marker (**Supplementary Figures 7A, B**). Finally, by using two co-localization indexes we revealed that GM-CSF co-localized with N-W at higher extent in tumors compared to the control surrounding tissues (**Figure 4C**).

Overall, our data indicated that a high rate of oxidative/nitrosative stress characterized the core of the human tumor lesion where the level of GM-CSF is elevated as compared to the normal epithelium.

## Discussion

Reactive oxygen species, including RNS that cause protein nitration/nitrosylation, are constantly generated in inflamed and transformed tissues as indicated by the high expression level of nitrosative stress markers in different human cancers (30). Our group previously discovered that the nitration of the inflammatory chemokine CCL2 limited T cell access into the tumor lesion by altering its chemoattractant properties (18). Nonetheless, the network of possible RNS-induced protein modifications within tumor specimens and the impact of such post-translational changes on the biology of the affected proteins can be expanded. In this study, we focused on a key cytokine - GM-CSF- that is abundantly secreted by several human tumors and plays a dual and still unclear role in cancer biology. Indeed, GM-CSF can act as a powerful stimulator of anti-tumor responses by favoring the recruitment and activity of NK cells, granulocytes, macrophages and antigen presenting cells in tumor-bearing hosts (31). Because of this rationale, GM-CSF has been proposed and employed as adjuvants in cancer immunotherapies. Clinical trials worldwide so far enrolled GM-CSF secreting tumor vaccines to treat patients with metastatic melanoma (31) prostate (32) and metastatic non-small-cell-lung cancer (33). Nevertheless, the clinical exploitation of this cytokine has showed conflicting results depending on cancer type and staging. Indeed in contrast with previous observations, it has been reported that high doses of GM-CSF can sustain cancer-related immunosuppression by increasing the number of immature myeloid cells (MDSCs) that actively suppresses anti-tumor T cell immunity (34). Later, *in vivo* evidence mechanistically connected GM-CSF to the intratumoral accumulation of highly immunosuppressive Gr-1^+^CD11b^+^ myeloid cells in a spontaneous pancreatic ductal adenocarcinoma (PDAC) model (12).

Taken together, all these findings indicated that the biology of the GM-CSF in tumors needs to be further expanded in order to reconcile the dual nature of this cytokine in cancer. Our study shed light on GM-CSF activity in tumors by indicating that this cytokine acts as a pliable element in cancer hosts. Indeed, we found that GM-CSF represents a new target for RNS in tumors. Furthermore, we investigated the functional significance of RNS-induced PTM for the biology of this cytokine. Specifically, by performing MS/MS analysis on *in vitro* peroxynitrite-treated protein we singled-out a specific aromatic residue, a tryptophan (W) which can be stably and consistently nitrated by RNS. The propensity of such residues to be modified by RNS was strengthened by a bioinformatic approach (homology modeling of the human GM-CSF) that clearly confirmed the accessibility of such residues for the interaction with radical species. Importantly, we found that RNS-modified GM-CSF can act as an amplifier of cancer-related immunosuppression by enhancing the generation and activity of myeloid suppressive cell subsets.

A key process occurring during tumor progression is the pathologic expansion and activation of distinct myeloid cell subsets that suppress adaptive immunity (35), collectively named MDSCs. Despite the considerable literature on MDCS classification, clinical studies have reported a positive correlation between infiltrating-myeloid cell percentage and stage progression in different human tumors as melanoma, advanced renal cancer (36), breast, bladder (37) and colon rectal carcinomas (38).

For this reason, MDSCs are now recognized as a worthy diagnostic marker and importantly as a key target for cancer immunotherapy. Our data showed that N-GM CSF promotes the expansion of PMN-MDSCs and macrophages, which represent highly immunosuppressive subsets.

The induction of MDSC phenotype and activity relies on multiple signaling pathways downstream cytokine stimulation, including GM-CSF. The canonical pathway triggered by the GM-CSF binding to its receptor involves primarily MAP kinases and STAT family members. In particular, STAT5 phosphorylation sustains the maturation of dendritic cells and it is commonly associated with pro-inflammatory responses. The duality in GM-CSF molecular signature is consistent with the complexity of cytokine-driven responses and it could be explained on the basis of GM-CSF dosage, signaling amplitude, and timing. Remarkably, we found that N-GM-CSF significantly amplifies the molecular signaling leading to MDSC expansion and activity compared to the native, unmodified GM-CSF. Our data indicated that N-GM-CSF can boost GM-CSF signaling through the STAT5 pathway. In tumor tissues where RNS are present at a very high extent (30) the post-translationally-modified cytokine competes with the native one for receptor binding. The selective ablation of N-GM-CSF so far could revert this balance by lowering the activation of immunosuppressive pathways thus promoting anti-tumor (pro-inflammatory) responses in tumor-bearing hosts. Importantly, our *in vivo* data confirmed that the nitration of GM-CSF represents a founding element for MDCS expansion and recruitment in tumor tissues. To deal with this issue, we engineered a fibrosarcoma cell line by introducing a single point-mutation in GM-CSF sequence, generating a tumor model that stably secretes a GM-CSF mutant which is insensitive to RNS-induced PTM. Indeed, the substitution of the site of RNS attack –a tryptophan- redraws the immune landscape at the tumor site. Tumors secreting the genetically-modified GM-CSF insensitive to RNS action (the W30L mutant) display a significant decrease in the number of both PMN-MDSC and macrophages (TAM) at the primary neoplastic foci. This occurs with comparable serum levels of the “native” *versus* mutated GM-CSF. For ethical reason we ceased tumor observation at day 21. We found no differences in the overall growth of tumor masses between tumor cell lines secreting either WT or W30L GM-CSF at this time-point. Nonetheless, the volume of W30L tumors was slightly decreasing from day 15, reasonably suggesting that the decreased recruitment of immunosuppressive subsets at the primary site could affect tumor growth on a longer timeframe. Consistently, the rate of nitrosivative stress was significantly decreased in W30L tumors compared to control as indicated by the reduced expression of both nitro-tyrosine and nitro-tryptophan markers.

The expansion and recruitment of PMN-MDCSs have been clearly connected to the promotion of cancer progression in different tumor models (39, 40) and human carcinomas (41). PMN-MDSCs can act in tumor bearing hosts by exploiting multiple mechanisms as the promotion of cancer-related angiogenesis (42) and the production of highly reactive species as peroxynitrite, O^2-^, and H_2_O_2_ (43). We also observed a trend in the decreasing of Mo-MDSC in W30L mice. At the tumor site, Mo-MDSC are rapidly committed to TAMs that historically exert a high ability to suppress T cell responses, in particular by producing nitric oxide and immune suppressive cytokines and expressing Arg1 (44).

Our data indicated that the nitration of GM-CSF represents a determinant element for the recruitment and expansion of both PMN-MDSC and TAM thus representing a promising target for immune-based therapy.

As largely reviewed (45), MDSCs originated from common BM precursors. Nonetheless, BM-MDCSs are poorly suppressive cells that need to exit this privileged site to exert their immunosuppressive activity. In line with this, MDCSs populating primary tumors and other sites as liver, blood and spleen can efficiently block T lymphocyte responses (40). In particular, the spleen represents an elective site for tumor-induced tolerance and remarkably splenectomy in tumor-bearing mice can reactivate anti-tumor immunity (27).

Of note, MDSCs isolated from the spleen of W30L GM-CSF tumor-bearing mice display a lower immunosuppressive activity compared to control WT tumors. Two enzymes participating to arginine-metabolism NOS2 and ARG1 are up-regulated in tumor specimens where act as key mediators for T cell suppressive mechanisms (7, 30). Importantly, the concomitant blockage of ARG1/NOS2 expression and activity harness MDSC ability to suppress T cell immunity (18). We found that NOS2 and ARG1 expression are significantly decreased in MDSCs isolated from W30L spleens, accounting for their reduced immunosuppressive activity in comparison to control cells. Indeed, our data confirmed that the nitration of GM-CSF nourishes the expansion, recruitment and activity of highly immunosuppressive myeloid cells both at the tumor primary site and systemically.

Along with the immune contexture, tumor vasculature plays a crucial role in dictating cancer progression and spreading. Indeed, growing tumors push the formation of new blood vessels to face their need of nutrients and oxygen supply. It is well established that oxidative/nitrosative stress promotes the generation of new vasculature sprouts within the tumor environment (46) representing so far a fascinating target for cancer therapy. In line with this, we observed that the reduction of the nitration rate in W30L tumor tissues goes with a significant decrease of the endothelial cell marker CD31 in the same specimens, thus suggesting a possible impairment of tumor angiogenesis in this context. The cross-talk between endothelial and immune cells is a relevant but still undefined issue in cancer biology. Indeed, angiogenic factors as VEGF-A and IL-8 contribute to the recruitment and expansion of distinctive immunosuppressive cell subsets as MDSCs and TAMs within the tumor environment (47). Mutually, MDSCs sustain tumor angiogenesis by the secretion of pro-angiogenic factors such as MMPs, VEGF and basic fibroblast growth factor (bFGF), or importantly by their direct embodiment into tumor-endothelia (48).

Cancer immunotherapy currently represents the most effective option for the treatment of patients with fatal cancers. A desirable achievement for immune-based treatments against cancer would be the definition of therapies that selectively harness anti-tumor immunity and overcome tumor immunosuppressive circuits. When we analyzed human colorectal specimens, we detected the presence of nitrated-W that colocalized with GM-CSF expression.

Although we are aware that colocalization approach does not provide clear-cut evidence for the presence of nitrated-GM-CSF within the tumor core, our findings suggested that the specific targeting of the nitrated-GM-CSF might represent an effective strategy to inhibit the tolerogenic activity of the modified protein in tumors without affecting the immunostimulatory properties of the native-unmodified cytokine.

Although further investigations are needed, our pre-clinical study pointed out nitrated- GM-CSF as a novel potential target for anti-cancer immune-based approaches.

## Data Availability Statement

The mass spectrometry proteomics data have been deposited to the ProteomeXchange Consortium *via* the PRIDE (19) partner repository with the dataset identifier PXD027049.

## Ethics Statement

This study was examined and approved by the Ethics Committee of the local Health and Social Services (Azienda Ospedaliera, Padova,n.57841, 3.12.2013) in accordance with the ethical standards laid down in the 1964 Declaration of Helsinki. The patients/participants provided their written informed consent to participate in this study. The animal study was reviewed and approved by the institutional protocols for animal care and approved by “Ministero della Salute”. All experiments followed committee guidelines CEASA 15/3/2012.

## Author Contributions

Conceptualization, BM and IM. Investigation BC, AA, CC, RS-R, AP, GIT, GA, FZ, CM, and LA. Writing- original draft, BM and IM. Critical data discussion, VB and AV. Funding acquisition, BM. All authors contributed to the article and approved the submitted version.

## Funding

Grant from the Italian Ministry of Health, BANDO GIOVANI RICERCATORI, No. 2009-GR-2009-1558698; Agnellini R.H. A. was granted by Cariparo Fundation Fellowship.

## Conflict of Interest

The authors declare that the research was conducted in the absence of any commercial or financial relationships that could be construed as a potential conflict of interest.

## Publisher’s Note

All claims expressed in this article are solely those of the authors and do not necessarily represent those of their affiliated organizations, or those of the publisher, the editors and the reviewers. Any product that may be evaluated in this article, or claim that may be made by its manufacturer, is not guaranteed or endorsed by the publisher.

## Abbreviations

BM, bone marrow; GM-CSF, granulocyte-macrophage colony-stimulating factor; IL-, interleukin; Mo-MDSC, monocytic MDSCs; TAMs, tumor-associated macrophages; MDSCs, myeloid derived suppressor cells; PMN-MDSCs, polymorphonuclear myeloid derived suppressor cells; PTM, post-translational modifications; RNS, Reactive Nitrogen Species; TANs, tumor associated neutrophils.
