# Supplementary material for: GM-CSF Nitration Is a New Driver of Myeloid Suppressor Cell Activity in Tumors
Source: Front Immunol. 2021 Oct 5;12:718098. doi: 10.3389/fimmu.2021.718098 (PMC8523982; doi:10.3389/fimmu.2021.718098)
Supplement: Supplementary file 1 [file DataSheet_1.pdf]

Figure 1S

A)

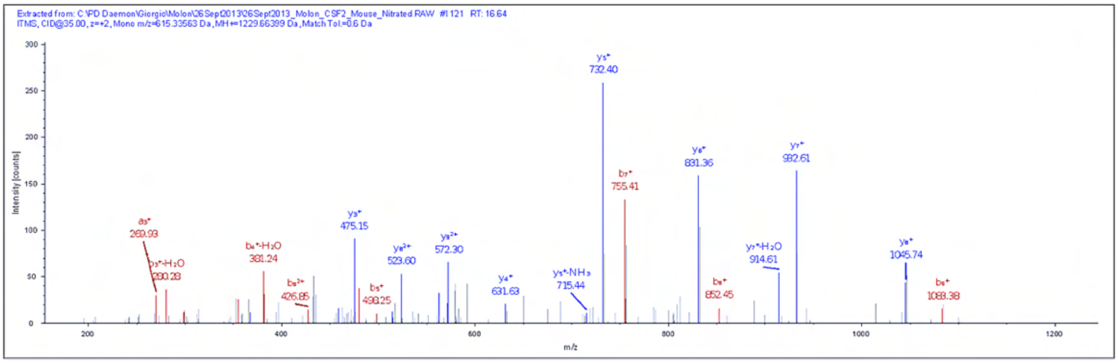

#1	a^+	a^{2+}	b^+	b^{2+}	Seq.	y^+	y^{2+}	#2
1	60.04	30.53	88.04	44.52	S			10
2	157.09	79.05	185.09	93.05	P	1142.63	571.82	9
3	270.18	135.59	298.18	149.59	I	1045.58	523.29	8
4	371.23	186.12	399.22	200.12	T	932.49	466.75	7
5	470.29	235.65	498.29	249.65	V	831.45	416.23	6
6	571.35	286.18	599.34	300.17	T	732.38	366.69	5
7	727.45	364.23	755.44	378.22	R	631.33	316.17	4
8	824.49	412.75	852.49	426.75	P	475.23	238.12	3
9	1055.56	528.29	1083.56	542.28	W-Nitro	378.18	189.59	2
10					K	147.11	74.06	1

Figure 1SA Annotated MS/MS spectrum relative to the doubly charged ion at m/z=615.3356 and corresponding to the nitrated peptide SPITVTRPWK (+44.9851 Da with respect to the unmodified peptide). In the Table, Mass difference between theoretical and experimental m/z is 0.54 ppm. Theoretical fragment ions matching the sequence are marked in red (for b and a series) and in blue (for y series).

Figure 1S

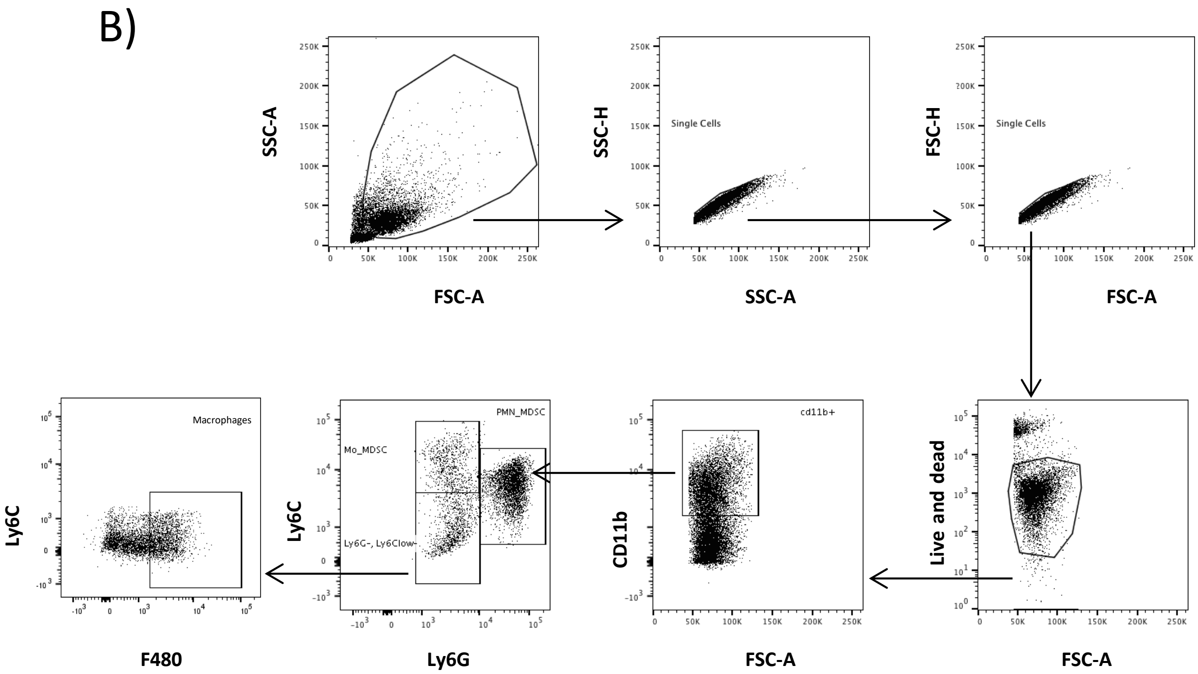

Figure 1SB *In vitro* MDSC gating strategy. Dot plots indicate the gating strategy of *in vitro* differentiated MDSC subsets.

Figure 2S

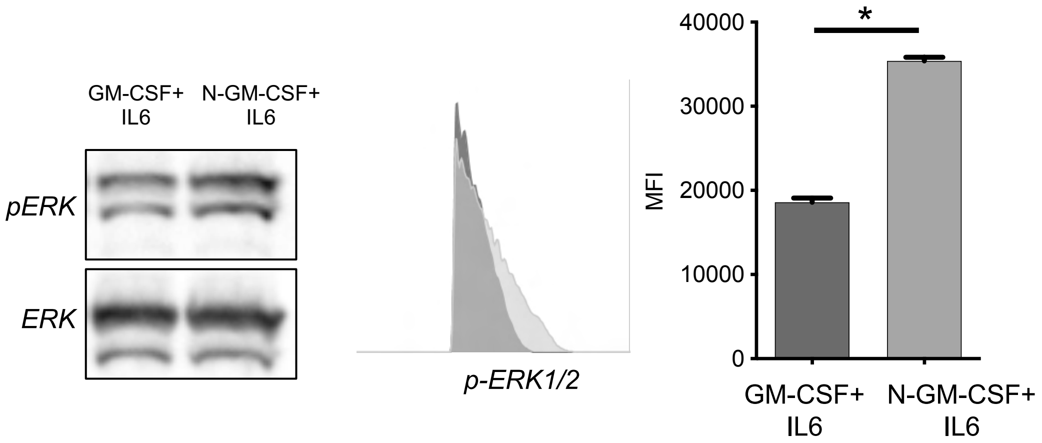

Figure 2S Representative WB and FACS analysis of the phosphorylation level (expressed as MFI) of Erk1/2 proteins in BM-MDSCs differentiated by GM-CSF or N-GM-CSF or in combination with IL-6.

Figure 3S

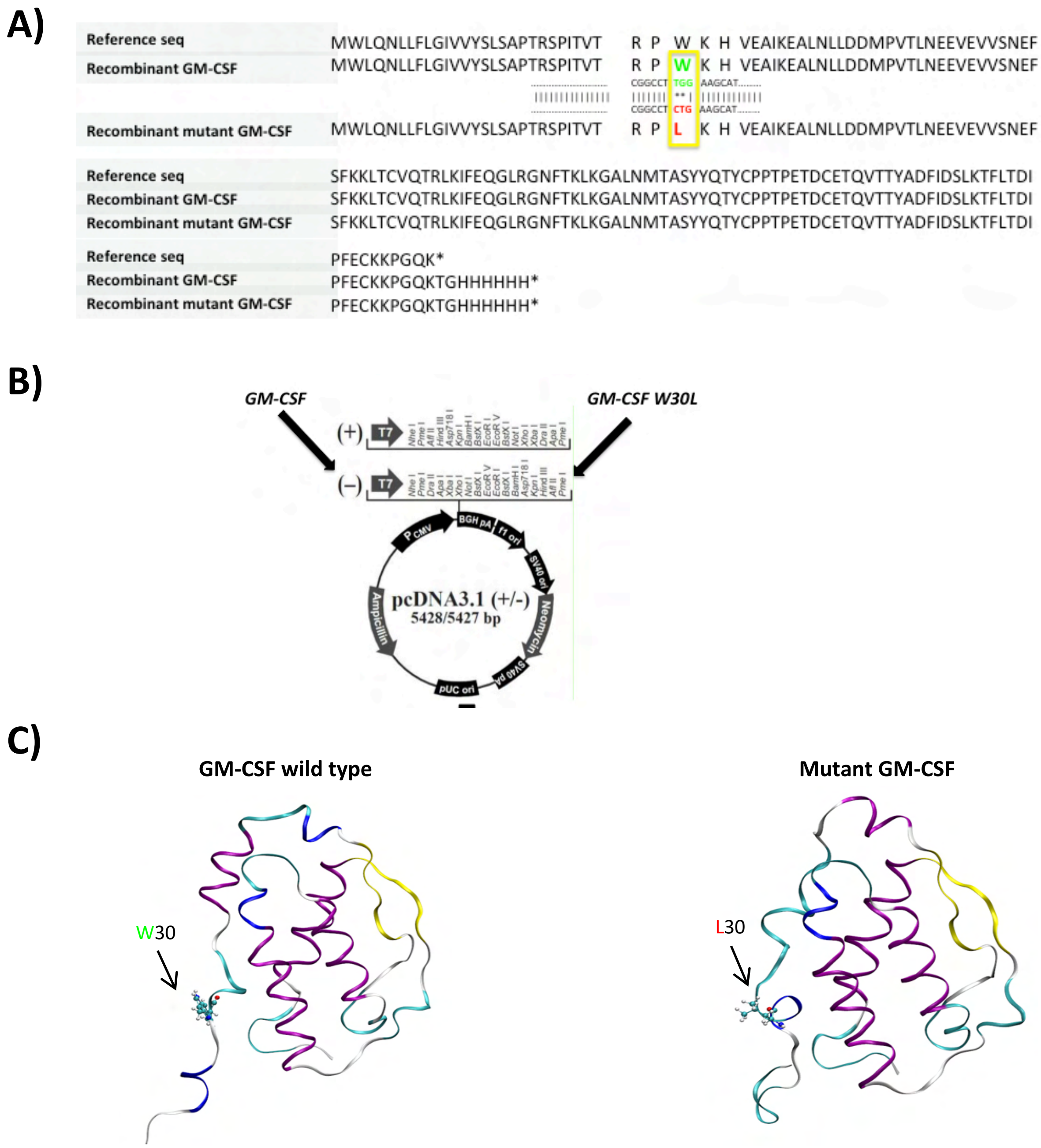

Figure 3S (A) Substitution in the sequence of GM-CSF of the aminoacidic residue ‘Tryptophan’, a target of nitration events, with a residue of ‘Leucine’, which is insensitive to nitration (Alvarez and Radi 2003) (B) Plasmids encoding either WT or nitration-resistant GM-CSF. Plasmids were used to enhance the secretion of recombinant GM-CSF in low-producing mouse tumor cell lines (MCA-203 fibrosarcoma). Transfection were optimized to achieve the highest efficiency (C) In silico analysis of GM-CSF compared with nitration-resistant GM-CSF. We replaced tryptophan with leucine: i) it is not known to be sensitive to nitration/nitrosylation; ii) it is present in amino acid sequences of orthologous and finally iii) it has the highest substitution score according two independent substitution

Figure 4S

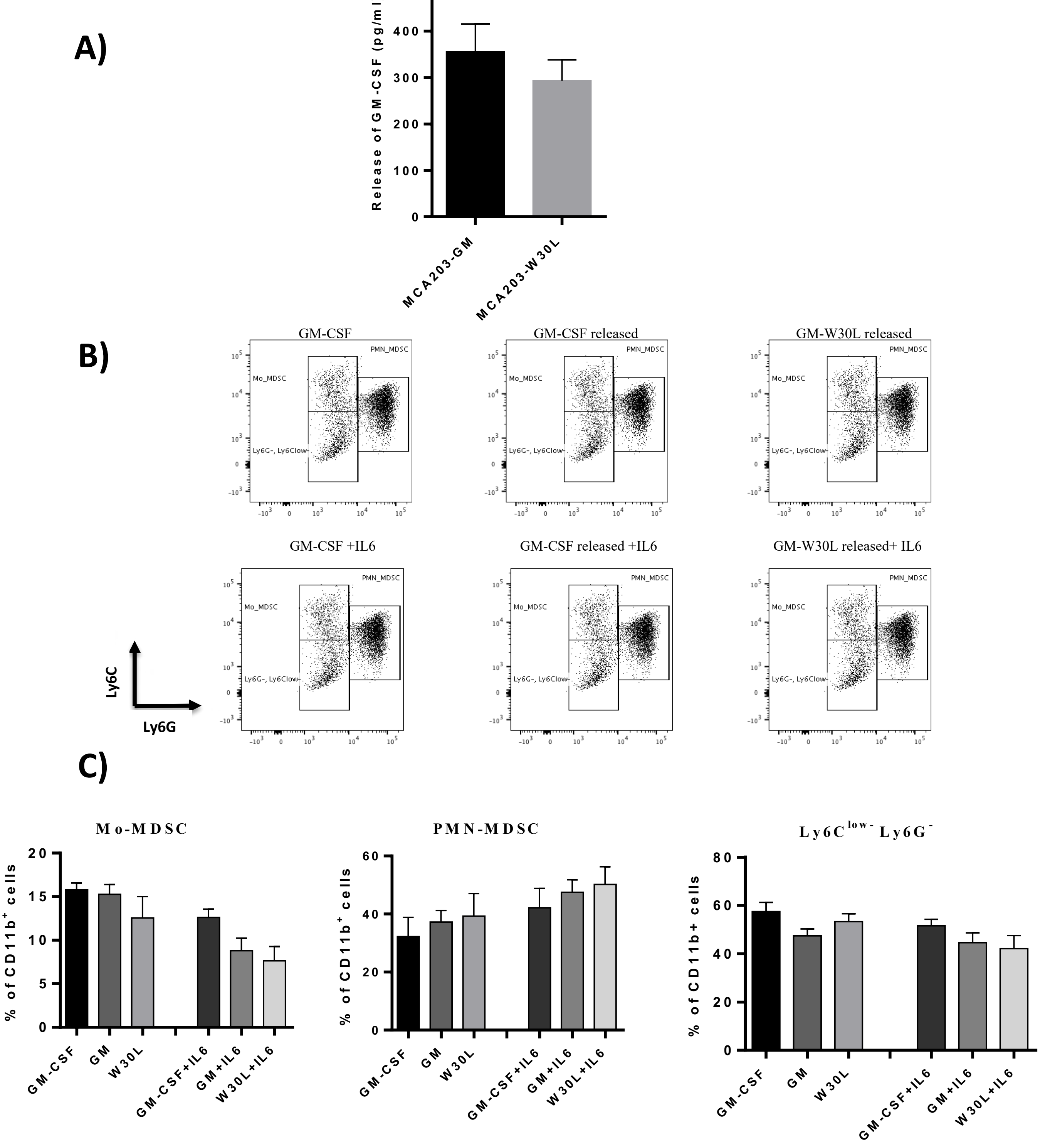

Figure 4S MCA-203 cells were stably transfected with plasmids encoding either WT (GM) or mutant GM-CSF (W30L). (A) Histograms represent GM-CSF quantification by ELISA in either WT (GM) or mutant GM-CSF (W30L) cell-culture supernatant. Conditioned cell media from either WT (GM) or mutant GM-CSF (W30L) culture were used to differentiate MDSC from BM-precursors. Recombinant GM-CSF alone or in combination with IL-6 was used as control for MDCS culturing. Representative dot plots (B) and quantitative analysis (C) of MDSC subsets after 5 days of culture. Data are representative of 3 independent experiments.

Figure 5S

A)

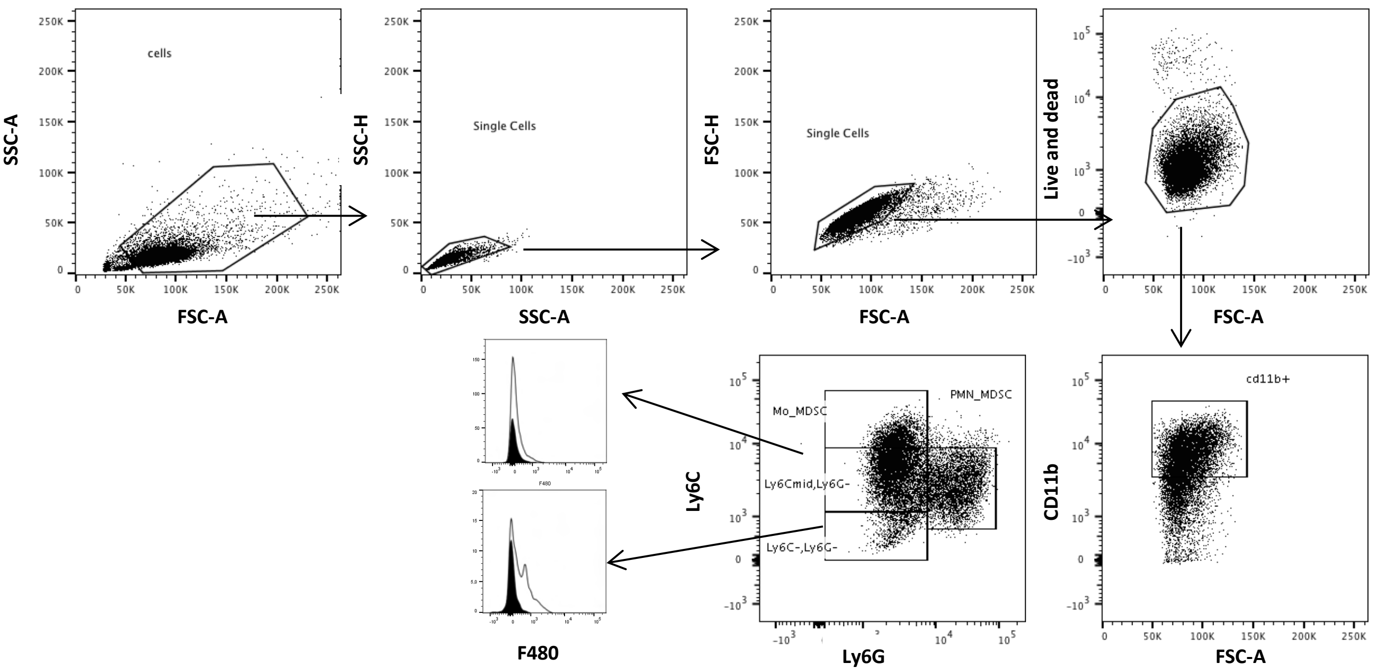

Figure 5SA *Ex vivo* MDSC gating strategy Dot plots indicate the gating strategy *ex vivo* MDSC subsets from either MCA203-GM or MCA203-W30L tumors.

Figure 5S

B)

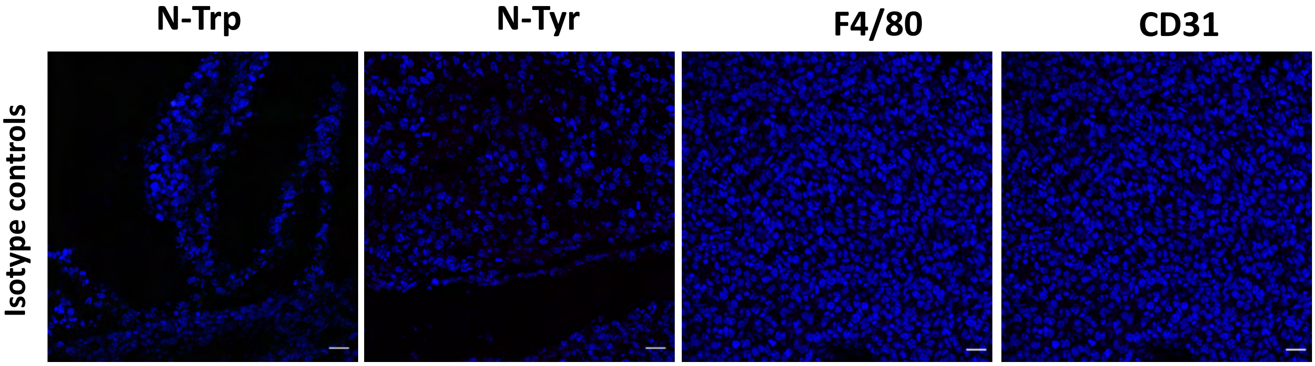

Figure 5SB Representative images of IF of tissue slices from either MCA203-GM or MCA203-W30L tumors. Tissues were stained with isotype control for nitro-tryptophan (grey), nitro-tyrosine (magenta) F4/80 (red) or CD31 (green). Scale bar 10 µm.

Figure 6S

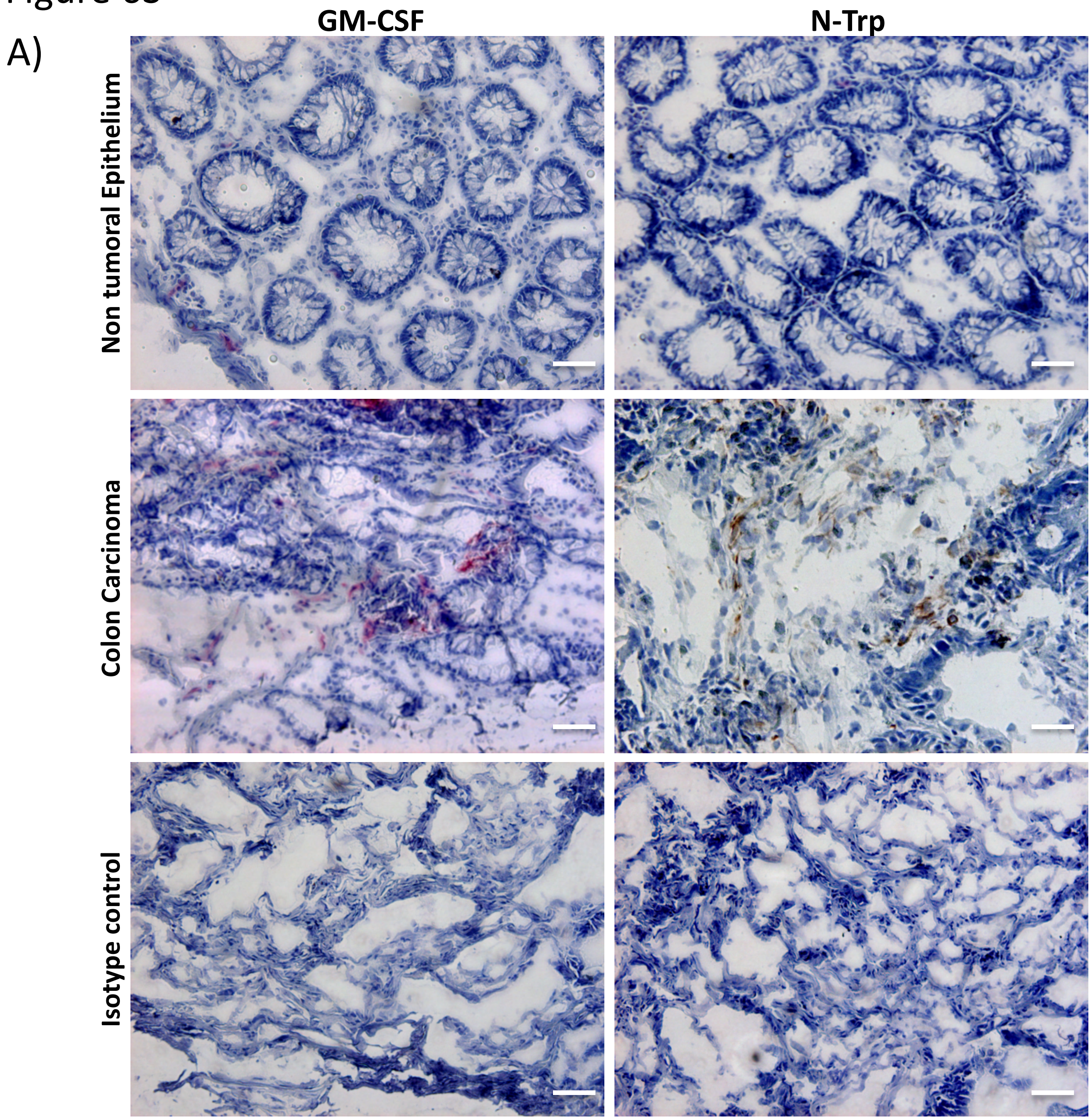

Figure 6SA Representative IHC serial sections of human colon carcinomas (carcinoma) or control colon tissues (NT-tissue) stained for GM-CSF (pink) or nitro-tryptophan (brown). Scale bar 20 μm.

Figure 6S

B)

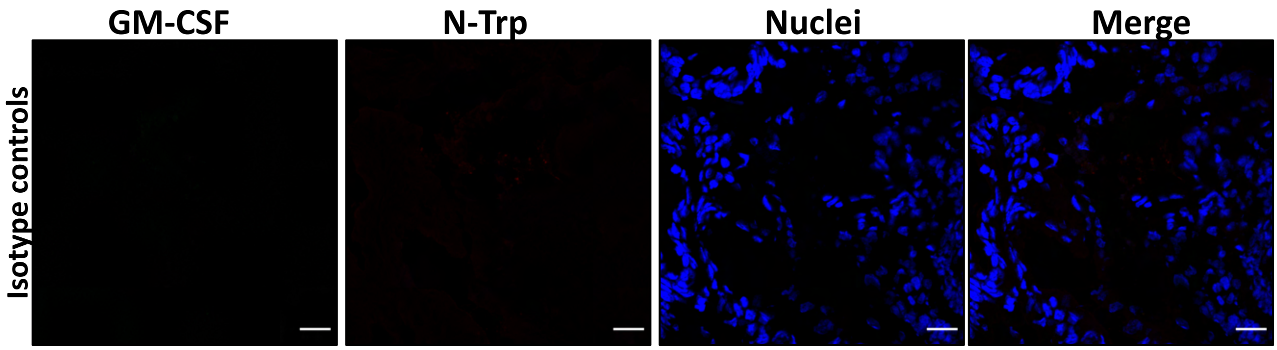

Figure 6SB Representative images of IF of tissue slices from in human colon carcinoma samples. Isotype control antibodies for green: GM-CSF (green) or nitro-tryptophan (red) scale bar 10 μm.

Figure 7S

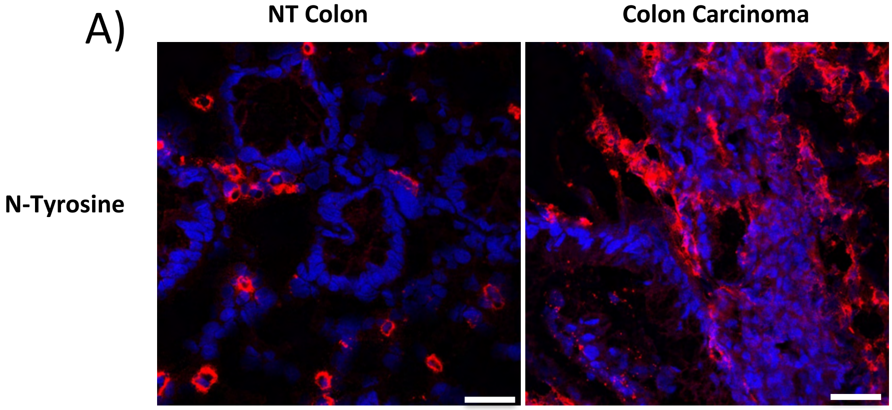

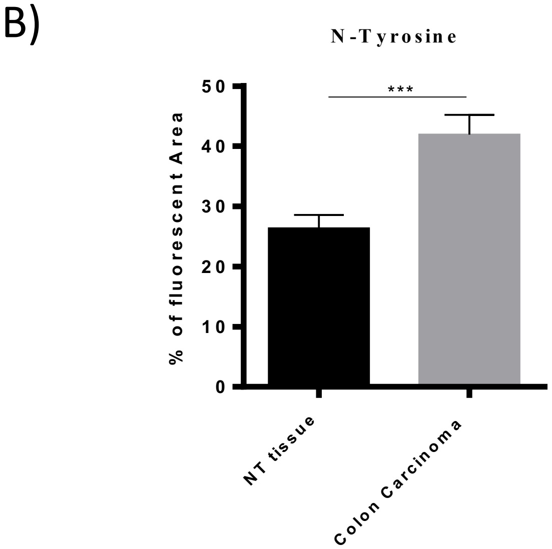

Figure 7S (A) Representative immunofluorescence images of serial sections of human colon carcinomas or non-neoplastic colon tissues, stained for nitro-tyrone (red); (B) quantification of fluorescence (positive reactive areas) from nitro-tyrosine staining. Data are representative of n=6 patient biopsies, unpaired Student T-test was performed ($p \leq 0.001$ =***). Scale bar 50 μm.
